# A Call for Systems Epidemiology to Tackle the Complexity of Schistosomiasis, Its Control, and Its Elimination

**DOI:** 10.3390/tropicalmed4010021

**Published:** 2019-01-29

**Authors:** Stefanie J. Krauth, Julie Balen, Geoffrey N. Gobert, Poppy H. L. Lamberton

**Affiliations:** 1 Institute for Biodiversity, Animal Health and Comparative Medicine, University of Glasgow, Glasgow G12 8QQ, UK; 2 School of Health and Related Research, University of Sheffield, Sheffield S1 4DA, UK; j.balen@sheffield.ac.uk; 3 School of Biological Sciences, Queen’s University Belfast, Belfast BT9 7BL, UK; G.Gobert@qub.ac.uk; 4 Wellcome Centre for Molecular Parasitology, University of Glasgow, Glasgow G12 8QQ, UK

**Keywords:** schistosomiasis, systems epidemiology, systems thinking, complexity, neglected tropical diseases, interdisciplinarity

## Abstract

Ever since the first known written report of schistosomiasis in the mid-19th century, researchers have aimed to increase knowledge of the parasites, their hosts, and the mechanisms contributing to infection and disease. This knowledge generation has been paramount for the development of improved intervention strategies. Yet, despite a broad knowledge base of direct risk factors for schistosomiasis, there remains a paucity of information related to more complex, interconnected, and often hidden drivers of transmission that hamper intervention successes and sustainability. Such complex, multidirectional, non-linear, and synergistic interdependencies are best understood by looking at the integrated system as a whole. A research approach able to address this complexity and find previously neglected causal mechanisms for transmission, which include a wide variety of influencing factors, is needed. Systems epidemiology, as a holistic research approach, can integrate knowledge from classical epidemiology, with that of biology, ecology, social sciences, and other disciplines, and link this with informal, tacit knowledge from experts and affected populations. It can help to uncover wider-reaching but difficult-to-identify processes that directly or indirectly influence exposure, infection, transmission, and disease development, as well as how these interrelate and impact one another. Drawing on systems epidemiology to address persisting disease hotspots, failed intervention programmes, and systematically neglected population groups in mass drug administration programmes and research studies, can help overcome barriers in the progress towards schistosomiasis elimination. Generating a comprehensive view of the schistosomiasis system as a whole should thus be a priority research agenda towards the strategic goal of morbidity control and transmission elimination.

## 1. A Brief History of the Discovery, Research, and Control of Schistosomiasis

The parasites now classified as *Schistosoma* spp. were first described in 1851 in Egypt by Theodor Bilharz [1]. Within a year, the involvement of these pathogens in the then-termed “endemic haematuria of warm climates” and the “dysenterische Veränderung des Dickdarms” (dysenteric pathology of the colon) became evident [1,2]. With estimated local prevalence ranging from 50–90% [3,4], and deaths from infections not uncommon, the disease initially named Bilharzia was recognised as a serious public health concern [3,4]. The parasite Bilharz first described as *Distomum haematobium* [1] was later renamed *Bilharzia haematobium* [3] and finally re-classified as *Schistosoma haematobium* [5]. Ever since Bilharz’s description, researchers have worked to understand the parasite’s developmental biology and identify strategies to help prevent infection, interrupt transmission, and reduce disease burden worldwide.

Bilharzia parasites were described in 1882 as “without question, the most dangerous [of human] parasite[s]” [3]. It was argued that, although other, more serious parasites existed, these were less common and therefore schistosomiasis should be considered the “schlimmerer Feind der Menschheit” (“worse enemy of humankind”) [4]. Attempts to control the disease were rolled out soon after its discovery and efforts have increased over the last two decades [6]. For example, one of the first schistosomiasis health education interventions was implemented in 1882. After recognising that infection takes place at water points, a printed memorandum was sent to employees of the British Eastern Telegraph company stationed in Egypt, and later made public [3]. In seeking to prevent human infection, a comprehensive understanding of the life cycle and mode of infection became important research priorities. Observations and experiments in the late 19th century led to hypotheses on how the parasite enters the human body, a point of biology debated in the literature of the time [3,4,7,8]. The most popular theory at the time suggested that ingestion of unclean water was the mode of infection [3,4], but skin penetration was also hypothesised early on and, although at first discounted, led to the policy of forbidding people to bathe in open waters [7,8]. Concurrently, efforts searching for an insect or mollusc intermediate host were ongoing due to analogies drawn from *Distoma* species [4].

Much progress has been made in the understanding of schistosomiasis since these early days. The biology of the parasites is now well described, intermediate host snails identified, and an effective antischistosomal drug—praziquantel—was developed in the 1970s, capable of treating infections caused by all species [9,10,11,12,13]. Nevertheless, Fritsch’s 1887 quote, “Even when we understand the life history [of Bilharzia] completely, we might not be able to sufficiently protect ourselves against this malicious foe” (translated from German) [4], remains true today, with over 200 million people still infected and over half of those demonstrating detectable symptoms [14].

Current international strategic goals, as outlined by the World Health Organisation (WHO), aim for morbidity control, and once this is reached for the elimination of schistosomiasis as a public health problem “where appropriate” by 2020 [6,15,16]. With such ambitious goals, it is important to consider how severe historical levels of schistosomiasis once were. If existing interventions were to be discontinued, interrupted, or otherwise unsuccessful, schistosomiasis prevalence, intensity, and associated morbidity could be at risk of returning to former historical levels [17,18].

Despite major research advances, many aspects of the biological, ecological, socio-cultural, economic, and political drivers of schistosomiasis are yet to be elucidated. To identify improved control measures, reduce disease transmission, and achieve elimination in the future, it is key to understand which factors and/or processes directly and indirectly influence exposure, infection, transmission, and disease development, as well as how these are interrelated. One priority area for research should therefore be to better understand, and engage with, the schistosomiasis “system” as a whole.

## 2. Systems Epidemiology: Systems Thinking for Epidemiology

To overcome current shortcomings of control efforts and move towards schistosomiasis elimination, there is a need to improve intervention strategies. It is well understood that health and disease are affected by multiple, diverse, and complex influences ranging from host immunology and parasite biology through to exposure, social environment, ecology, climate, and access to preventative and curative services [19,20,21,22,23]. Control strategies need to be designed around an improved understanding of the comprehensive range of broader factors influencing disease transmission and intervention successes. Their complex interrelationships, emergent properties, and non-linear feedback-loops all need to be considered. Such interdependencies in a system cannot be identified by considering each of the factors individually. These relationships are best understood by looking at the system as a whole [20,23,24]. Traditional epidemiological approaches, largely reductionist in nature, deal with a limited number of directly related factors, narrowing down causal mechanisms to smaller components that enable us to draw generalisable conclusions [25,26]. This approach has been paramount to uncover many risk factors of *Schistosoma* transmission. However, the direct and indirect interrelated causal mechanisms of the disease are hard to integrate using standard epidemiological approaches. In contrast to reductionist approaches, systems sciences address system-wide behaviours and collective effects. In systems thinking, a system is understood as consisting of many components that, through their interactions with each other, form a complex whole with system-wide properties that can give rise to emergent behaviour, adaptations, and feedback loops [27].

The application of general systems thinking to epidemiology and health research as a tool to integrate knowledge from different areas of research has long been proposed for several diseases [19,28,29,30]. However, to our knowledge this is the first time it has been proposed specifically for schistosomiasis or any neglected tropical disease (NTD). Systems thinking has well-defined advantages over reductionist approaches. A good example of a health issue of which we now have a system-wide understanding is obesity [24,28,29]. A system influence diagram created by Shiftn (2008) illustrates the complex interrelationships of multiple factors influencing obesity [28]. Such “influence models” can be used to formulate appropriate research hypotheses and address the multiple factors across levels of influence and across disciplines [23,26,29]. With influence diagrams, the whole-disease system can be effectively visualised and analysed for underlying causal mechanisms driving changes in infections and intervention successes/failures. This type of analysis can provide the starting point for more in-depth studies on the dynamics of human–parasite systems as well as for more contextually-relevant intervention/implementation research. Co-creating and discussing influence diagrams can, in turn, enhance stakeholders’ understanding of underlying behaviours of a specific system [24]. 

In their thematic series, “Advancing the application of systems thinking in health” Adam (2014) and colleagues discussed how the application of various systems methods has helped authors uncover reasons for poor health outcomes, such as systems-wide impacts on neonatal health in Uganda and has gone on to identify high leverage points [30]. Other authors linked a range of factors including government opposition, alternative medicine, and strong media coverage to changes in vaccination rates [31]. Adam (2014) emphasised the importance of including evidence beyond traditional epidemiology and economic analysis into the design and evaluation of public health interventions and discussed the usefulness of visual representations for the analysis and interpretation of a system [30]. We build on these ideas by proposing the integration of systems science tools and systems modelling into the field of epidemiology for schistosomiasis and other NTDs. This approach, described as “systems epidemiology”, will more effectively integrate knowledge to better understand and control schistosomiasis, as well as focus on contextually relevant factors. 

The central goal of a systems epidemiologist would be to uncover principles governing the behaviour and outputs of the entire system, not limited to the behaviour of its individual parts. Working together in a trans-disciplinary manner, researchers, policymakers, health providers, and the affected population could generate much-needed insights into the drivers of persisting *Schistosoma* transmission.

## 3. Systems Epidemiology as Described in Broader Existing Literatures

Systems epidemiology has, to the best of our knowledge, not yet been applied in the form we propose here. However, the term has been used several times in recent publications across a range of scopes and other topics [19,32,33,34]. In the majority of these studies, systems epidemiology refers to integrating systems biology tools, such as high-throughput molecular analysis of biomarkers, with epidemiological research questions. For example, the integration of genomics, transcriptomics, proteomics, and metabolomics with epidemiological research was conducted to clarify underlying mechanisms of the effects of food on human health [32]. In addition, a multi-omic approach was employed to understand human pathophysiology in cardiovascular disease and cancer [33,34]. Including multi-omic measurements to analyse complex biological data is an important strategy to uncover the exact mechanisms involved in exposure through to disease development. However, systems biology comprises only the micro-system that influences disease development; i.e., mechanisms only taking place after host exposure to the parasite. In contrast, systems epidemiology can be defined in a wider context, whereby mechanisms related to environmental, social, and demographic aspects of disease are needed to complement the systems biology data, as previously proposed for the control of tuberculosis [19].

We therefore argue that systems epidemiology needs to move beyond the application of systems biology, towards a more holistic understanding of health. Systems epidemiology should combine classical epidemiology with social sciences, natural sciences (including systems biology), ecology, economics, health policy and systems research, and other relevant disciplines and sub-disciplines. This would more effectively characterise the relevant physical and socio-political environments of endemic regions, and access (or lack of) to relevant services, as well as the biology and the co-evolution of the hosts and the pathogen. We argue that applying a systems epidemiology approach to schistosomiasis research is imperative for assessing factors that contribute to persistent disease hotspots, failed intervention programmes, and systematically missed population groups in mass drug administration (MDA) campaigns and/or research studies. Findings from such an approach, we believe, can help overcome current barriers in the progress towards schistosomiasis elimination.

## 4. The Call for Systems Epidemiology Approaches in Schistosomiasis Control

Over the last two decades, the global agendas outlined in the Millennium and Sustainable Development Goals (MDGs and SDGs, respectively) have raised the profile of schistosomiasis and other poverty-related diseases which had, in the post-colonial era, received reduced attention by international and national governmental agencies [35]. The WHO recommends repeated MDA with praziquantel to reduce schistosomiasis intensity and associated morbidity [14]. In certain areas where the elimination of schistosomiasis as a public health problem, or the interruption of schistosomiasis transmission, are deemed possible, the WHO recommends MDA plus complementary public health interventions [14]. MDA reduces the number of *Schistosoma* eggs released into the environment, thus reducing human to snail transmission and potentially lowering transmission back into humans [14,36,37]. MDA campaigns have made substantial reductions in morbidity rates and have improved public health overall [37]. These successes sparked an increased push towards disease elimination [6,15,16]. However, MDA success varies considerably, with greater reductions in infection intensities and prevalence in areas which were initially classified as low endemicity (<10% of school-aged children infected), but with often disappointing effects in many moderate and high endemicity areas [21,38]. Despite increased funding, extensive praziquantel donations and national control programmes running for over a decade in several countries, hotspots of high transmission and severe morbidity remain [21,38,39].

While the benefits of MDA to schistosomiasis morbidity reductions are well established, it is now evident that MDA alone is unlikely to achieve elimination [21,36,37,40,41]. Many authors have discussed reasons for MDA programme failures, including treatment compliance, inadequate coverage, treatment efficacy, open defecation/urination behaviours, water contact behaviours, snail density, available infrastructure, and many more [21,36,40,42,43,44,45,46,47]. Because schistosomiasis is so deeply embedded in broader physical, social, political, and economic systems, the factors influencing intervention successes (or failures) also span these systems [40,42,45]. Several integrated control measures have been proposed to overcome currently identified shortcomings of MDA-only strategies. These propose the inclusion of health education, agricultural policy interventions, sanitation improvements, water supply improvements, engineering interventions, snail control, and behavioural interventions, etc. [48,49,50,51,52,53]. Extending control measures in this way aims to target both the transmission of schistosomiasis from snails to humans and from humans to snails. Most of these measures aim to increase treatment compliance, provide physical barriers around urine or faeces, and/or decrease snail density. The proposed measures in these studies are based on factors known to contribute to transmission and disease burden. However, a comprehensive summary of all prevailing factors influencing the disease and its transmission in specific settings, and how changing one aspect impacts another aspect as well as the overall system, has, to date, not been generated. Due to the complexity of the issues, it is unfeasible to identify, measure, and include all factors in a single study and researchers often need to focus on a subset of relevant factors that are measurable and feasible for analysis. A research approach and associated toolkit able to deal with this complexity, including a variety of influencing factors, is urgently needed if WHO goals are to be met.

## 5. How to Apply Systems Epidemiology Approaches to Schistosomiasis Control

**Figure 1:**
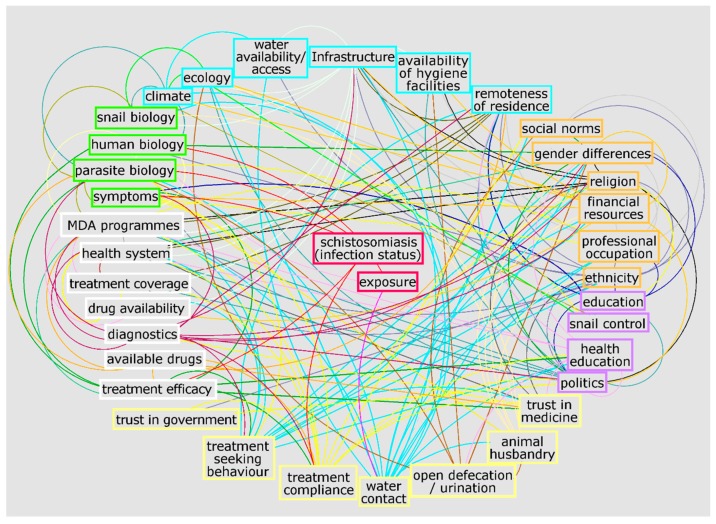
Potential network of factors influencing schistosomiasis infection and disease created with Vensim [59] and GIMP [60]. Connections are a collection of empirically tested or hypothesised relationships. Colours are for illustrative purposes only. This diagram resulted from ongoing brainstorming of connections from a large body of literature and expert knowledge. The diagram aims to exemplify the complexity of the schistosomiasis system without claims of completeness and without detailing the exact nature and direction of interactions. It is meant solely for the purpose of an example of what a systems diagram for schistosomiasis could provide. The full diagram(s) would need to be developed through extensive empirical and theoretical research.

**Figure 2:**
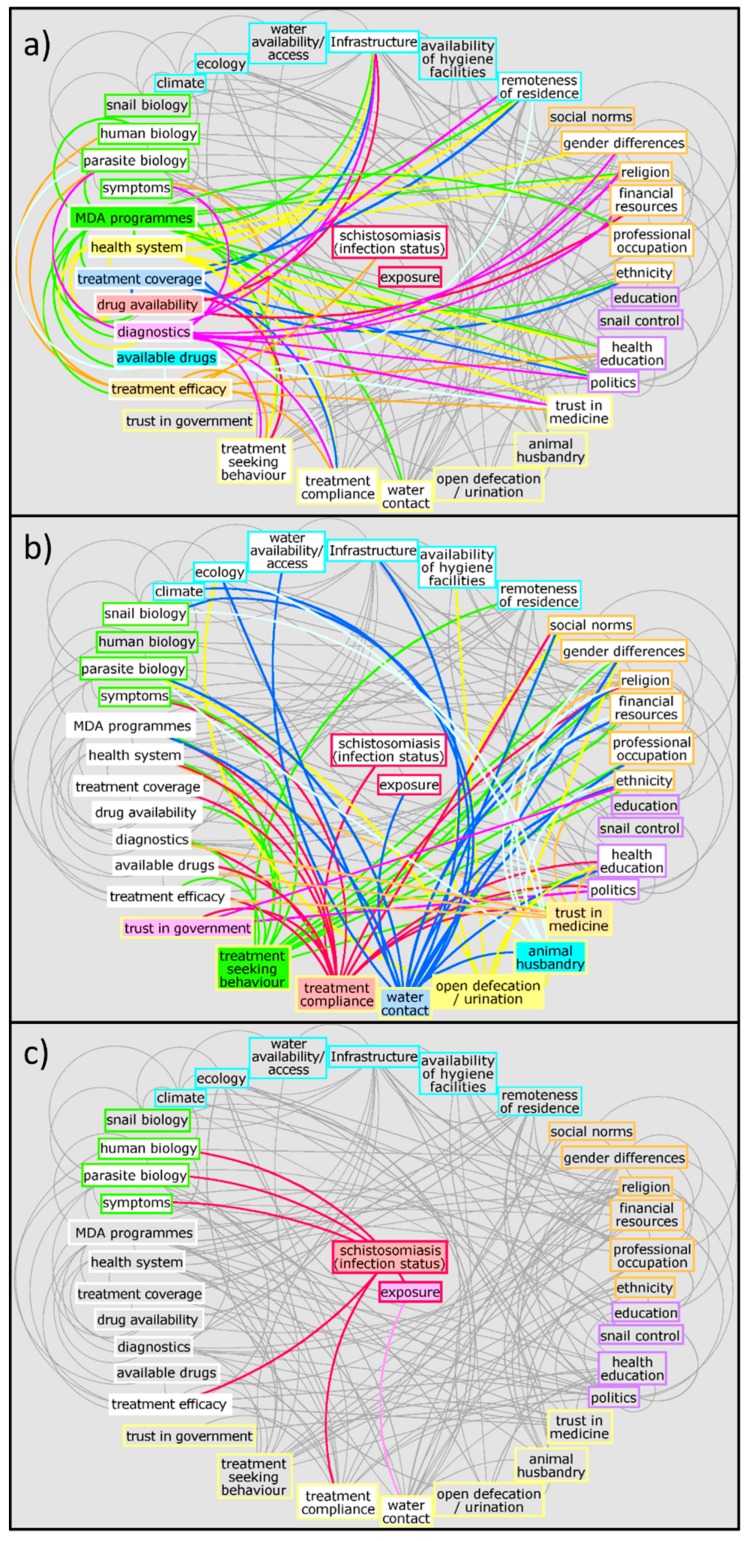
Potential network of factors influencing schistosomiasis infection and disease with individual connections highlighted for (**a**) clinical, (**b**) behavioural, and (**c**) exposure and infection aspects. Colours are for illustrative purposes only; white boxes indicate where the highlighted variables connect to. Different clusters are highlighted by coloured outlines: green: biological aspects; white: clinical aspects; yellow: behavioural aspects; orange: social aspects; purple: politics, policy, and services aspects; turquoise: physical environment; red: exposure and infection.

**Figure 3:**
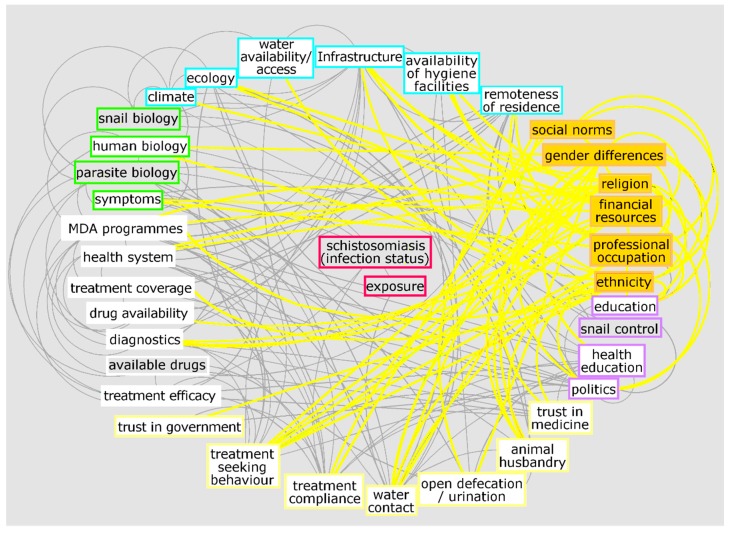
Interconnectedness of social factors with other aspects of the schistosomiasis. White boxes indicate where the highlighted variables connect to. Social aspects illustrated: social norms, gender differences, religion, financial resources, professional occupation, and ethnicity. Although they do not interact directly with infection status or exposure, social aspects underlie and influence many other factors that are, in turn, connected to exposure and infection.

To exemplify the complexity of the schistosomiasis system, Figure 1 and Figure 2 illustrate a preliminary interrelationship diagram of empirically tested or hypothesised interactions from a large body of literature and expert knowledge. These figures represent a first “brainstormed” draft of the broader interaction network for schistosomiasis that could be created using systems epidemiology tools. Detailing the complete nature and direction of these interactions would take considerable future research. Nevertheless, already from this crude diagram, interesting observations emerge. For example, factors from the social environment are especially well-connected to other factors in the system (Figure 3). While social environment factors rarely directly interact with infection mechanisms, they are connected with other factors which, in turn, influence exposure and/or treatment coverage and thereby infection rates of a community. These factors affect, e.g., the likelihood of having high-risk water contact, access to treatment, and treatment compliance—which are all well-documented MDA programmatic issues [21,47,54]. Social environment factors vary between communities and settings, since social and cultural norms vary between groups [55,56,57]. Additionally, social environment factors are not easily quantifiable and often need qualitative research approaches to elucidate them. However qualitative research remains neglected and still occasionally overlooked or misunderstood. This is perhaps best exemplified in the 2016 systematic review on treatment compliance in NTD control programmes, in which qualitative findings were discussed in half a sentence noting: “Other studies provided only qualitative or anecdotal data or reasons for low or non-compliance” [47]. Qualitative research is especially valuable and urgently needed when assessing treatment uptake in order to find missing factors to explain low compliance. Complete whole-system diagrams could further be compared with other, more commonly used methods, such as Bayesian networks [58], to compare combined qualitative and quantitative approaches.

Understanding schistosomiasis control as a systems issue requires a strong interdisciplinary and transdisciplinary approach. Strategies are needed to enable researchers and programme managers to adequately assess and integrate issues across disciplines and in different settings. Creating an empirically based system for understanding interrelationships between relevant issues in schistosomiasis transmission and intervention successes/failures is needed. A comprehensive interrelationship diagram, detailing the nature and direction of the system-wide connections would need to be based on the integration of published knowledge from researchers, with informal knowledge from experts and local populations. Methods to collect and analyse data would include repeated modelling sessions and participatory workshops with stakeholders from a range of backgrounds. This would be the first step towards establishing a systems epidemiology research approach for the control and elimination of schistosomiasis and other NTDs.

## 6. Conclusions

Understanding the broader systems-relations and influences of a disease, as well as how best to address these factors with limited resources, is an important challenge. The overall goal is to establish sustainable, contextually relevant, and cost-effective approaches to tackle persistent hotspots of disease transmission. Moving beyond current research and implementation practice, towards complex system analyses and interventions for a disease such as schistosomiasis will help research and policy communities identify how best to achieve WHO goals along the road to elimination. Using a systems thinking toolset, we can connect and integrate knowledge from across a range of disciplines including classical epidemiology, molecular parasitology, ecology, medical anthropology, social and political sciences, and health policy and systems research to identify multiple target points for future schistosomiasis control programmes. This would remove or minimise elusive barriers to success, enabling the global health community to move a step closer towards the elimination of schistosomiasis worldwide.
